# Sequential deletion of genes from the African swine fever virus genome using the cre/loxP recombination system

**DOI:** 10.1016/j.virol.2012.07.021

**Published:** 2012-11-10

**Authors:** Charles C. Abrams, Linda K. Dixon

**Affiliations:** Institue for Animal Health, Pirbright Laboratory, Ash Road, Pirbright, Woking, Surrey, GU24 0NF, United Kingdom

**Keywords:** ASFV African swine fever virus, Cre/loxP recombination, Recombinant virus

## Abstract

A method has been established to sequentially delete combinations of genes from the ASFV genome to test the effect on virus replication and host responses to infection. Initially the ASFV genes MGF505 2R and MGF505 3R and a truncated MGF360 9L gene were deleted from the genome of the tissue-culture adapted ASFV strain BA71V and replaced with bacteriophage loxP sequences flanking the beta-glucuronidase (GUS) marker gene to create recombinant virus VΔMGF-GUS. Subsequently the GUS gene was removed by site-specific recombination between the two loxP sites involving expression of the bacteriophage Cre recombinase enzyme to create recombinant virus VΔMGFΔGUS. The EP402R and EP153R genes were subsequently deleted from the genome of VΔMGFΔGUS, using the same GUS marker gene, to construct virus VΔMGFΔCD2-Lectin-GUS. These sequential deletions of ASFV genes were shown not to alter virus replication significantly.

## Introduction

ASFV causes a devastating haemorrhagic disease of domestic pigs that was first described in East Africa in the 1920s. The virus is present in that region in a wildlife cycle involving warthogs and soft ticks of the *Ornithodoros* species. In these hosts, the virus causes persistent infections with few disease signs. The disease is endemic in many sub-Saharan African countries and causes sporadic outbreaks in others. Following its introduction to Georgia in 2007, ASF has now spread extensively within the Russian Federation and has been reported within 100 miles of the EU border. Currently there is no vaccine available against ASFV. Virulent isolates kill domestic pigs within 7–10 days of infection, with a mortality rate approaching 100%. However, less virulent strains do not necessarily kill and recovered pigs can be immune to subsequent challenge with related virulent viruses.

ASFV is a large, icosahedral, cytoplasmic virus and is the only member of the family *Asfarviridae* (Dixon et al., 2005). The virus has a linear double-stranded DNA genome varying from 170 kb to 194 kb depending on the isolate (Chapman et al., 2008, 2011; De Villiers et al., 2010). The complete coding sequences of the Vero cell-adapted strain, BA71V (Yanez et al., 1995), and of several high and low virulence isolates of ASFV have been reported (Chapman et al., 2008, 2011 and De Villiers et al., 2010). These reports have identified genes that are not present in low virulence isolates compared to high virulence isolates including a sequence close to the left genome end of about 8 kb containing six copies of multigene family (MGF) 360 and two of MGF 505. Deletion of these genes from the genome of a virulent isolate was shown to result in increased production of type I interferon (Afonso et al., 2004). The low virulence OURT88/3 isolate also has frame-shift mutations in the EP402R and EP153R genes. These encode the CD2v protein and C-type lectin protein, respectively (Chapman et al., 2008). CD2v is required for the binding of red blood cells to extracellular virus and infected cells (Rodriguez et al., 1993; Borca et al., 1994). This protein has also been indicated to have a role in the impairment of lymphocyte proliferation in response to mitogens (Borca et al., 1998). Expression of the C-type lectin protein has been shown to inhibit up-regulation of cell surface expression of MHC Class I molecules (Hurtado et al., 2011) but its deletion does not affect virus growth in macrophages or virulence in swine (Neilan et al., 1999; Galindo et al., 2000).

The role of ASFV encoded proteins has been investigated by deletion of genes from the virus genome. In common with other large DNA viruses, ASFV is known to encode several proteins which target the same pathways. Therefore to investigate the role of particular pathways in the virus interaction with its hosts, it would be advantageous to delete several different genes.

Methods for making gene deletions within the ASFV genome using homologous recombination to replace a specific gene with a reporter gene were first established by Rodriguez et al., 1992. Subsequently the β-glucuronidase (GUS) gene was used as a reporter to select recombinant field isolates grown in primary pig macrophages or COS-1 cells (Zsak et al., 1996). These methods were successfully used to make single gene deletions including individual genes DP71L, DP96R, CD2v and A238L or several adjacent genes from virulent strains including Malawi LiL20/1, E70 and Pr4. (Afonso et al., 1998; Zsak et al., 1998; Borca et al., 1994 and Neilan et al., 1997). However, once the marker gene has been inserted, this same marker gene cannot be used to make a second deletion. In order to overcome this we used the bacteriophage cre/loxP recombination system to efficiently delete the marker gene from a first generation virus recombinant and subsequently re-used the same marker gene to isolate a virus gene deletion at a second locus.

The cre/loxP recombination system was first discovered in the bacteriophage P1 by Sternberg and Hamilton, 1981 and demonstrated to operate in mammalian cells by Sauer and Henderson 1988. This system involves the specific deletion of a DNA sequence located between two bacteriophage loxP sites and is controlled by the bacteriophage Cre recombinase enzyme. Here we describe the construction of transfer vectors containing loxP sites flanking the marker gene GUS under the control of the ASFV B646L (VP72) gene promoter. Recombinant ASF viruses lacking specific genes were obtained by insertion of this GUS marker gene by homologous recombination to replace the genes of interest. First generation recombinant viruses deleting a truncated copy of the MGF 360 9*L* and the complete MGF505 2*R* and 3*R* genes were isolated by screening for expression of GUS. Subsequently the GUS marker gene was deleted using the Cre recombinase to produce a second generation recombinant virus lacking the GUS marker gene. The GUS marker gene flanked by loxP sequences was then reinserted into a second locus with the deletion of the ASFV adjacent genes EP153R and EP402R to create a third generation virus. Finally the GUS marker gene was deleted from this virus using the cre/loxP recombination system.

This cre/loxP recombination system will enable sequential deletion of genes from the ASFV genome to facilitate studies on their individual protein functions. In addition, recombinant viruses containing genes deleted in different combinations will allow us to study the host response to potential new vaccines.

## Results and discussion

### Deletion of the MGF505 2*R* and 3*R* genes and truncated MGF 360 9*L* gene from the BA71V ASFV isolate

**Fig. 1:**
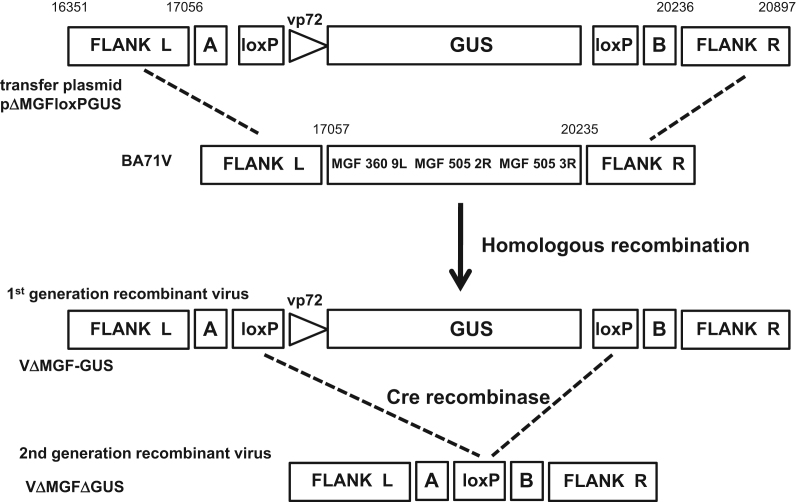
Schematic diagram showing generation of recombinant ASF viruses expressing GUS reporter gene and excision of the GUS gene by the bacteriophage Cre recombinase. First generation recombinant virus VΔMGF-GUS was created by homologous recombination between left and right flanking regions (FLANK L and FLANK R) from genes MGF360 8*L* and MGF 505 4*R* on the wild type BA71V genome and transfer vector plasmid pΔMGFloxPGUS resulting in the deletion of the truncated MGF360 9*L* and MGF505 genes 2*R* and 3*R* and the insertion of vp72GUS gene flanked by loxP sequences. A plasmid expressing the bacteriophage Cre recombinase protein under control of the ASFV vp72 gene promoter was transfected into cells infected with VΔMGF-GUS and second generation virus recombinant VΔMGFΔGUS was generated by site-specific recombination between the two loxP sites resulting in deletion of the vp72GUS marker gene and single loxP site and the retention of a single loxP site. Non viral sequences A and B were included to facilitate the construction of plasmid pΔMGFloxPGUS by overlapping PCR.

A transfer plasmid, pΔMGFloxPGUS was constructed (see the Materials and methods section) to enable the truncated MGF360 9*L* and MGF505 2*R* and MGF505 3*R* genes to be deleted from the genome of the BA71V isolate by homologous recombination and be replaced with the GUS gene (Fig. 1). This plasmid contained fragments from the genes flanking the deletion site; MGF360 8*L* to the left and MGF505 4*R* to the right. It also included loxP sites flanking the GUS reporter gene under control of the ASFV B646L (vp72) late gene promoter. The loxP sites were inserted to subsequently facilitate excision of the GUS reporter gene from the ASFV genome using the Cre recombinase protein. Short unique sequences, named A (21 bp) and B (23 bp), were included to facilitate overlapping PCR amplifications used for plasmid construction. The method to generate the vector pΔMGFloxPGUS by overlapping PCR amplification is described in the Materials and methods section.

**Fig. 2:**
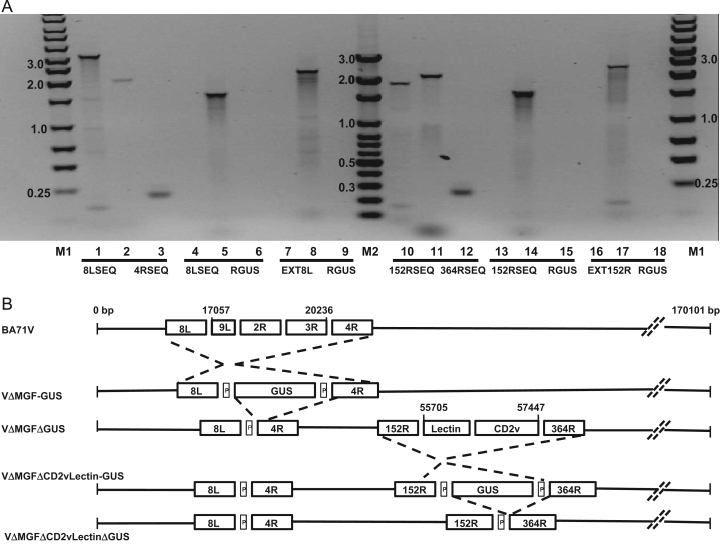
Generation of successive recombinant ASFV viruses Panel A. Analysis of genomic viral DNA gene deletions and insertions by PCR. Viral DNA was extracted from wild type BA71V virus and the recombinant viruses VΔMGF-GUS, VΔMGFΔGUS, VΔMGFΔCD2vLectin-GUS, VΔMGFΔCD2vLectinΔGUS. Specific fragments were amplified by PCR and the products were analysed by electrophoresis on 1% agarose gels. The following primer sets were used in the lanes 1–3 (8LSEQ+4RSEQ, lanes 4–6 (8LSEQ+RGUS), lanes 7–9 (EXT8L+RGUS), lanes 10–12 (152RSEQ+364RSEQ), lanes 13–15 (152RSEQ+RGUS), lanes 16–18 (EXT152R+RGUS). The following viral genomic DNAs were used as templates in the lanes 1, 4, 7, 10, 13 and 16 (BA71V), lanes 2, 5 and 8 (VΔMGF-GUS), lanes 3, 6 and 9 (VΔMGFΔGUS), lanes 11, 14 and 17 (VΔMGFΔCD2vLectin-GUS), lanes 12, 15 and 18 (VΔMGFΔCD2vLectinΔGUS). Lane M1 contains a 1 kb DNA ladder and lane M2 a 100 bp DNA ladder. Panel B. Genomic maps of BA71V (170101 bp) and the four recombinant viruses showing the positions of deletions of the truncated MGF360 9GL, MGF505 2*R* and 3*R* genes located between positions 17725 and 20236. The two recombinant viruses VΔMGFΔCD2vLectin-GUS and VΔMGFΔCD2vLectinΔGUS show the additional deletion of the EP153R and EP402R genes located between positions 55,705 and 57,447. Recombinant viruses VΔMGF-GUS and VΔMGFΔCD2vLectin-GUS show insertion of the GUS gene flanked by two loxP (P) sequences and viruses VΔMGFΔGUS and VΔMGFΔCD2vLectinΔGUS show deletion of the GUS gene retaining one copy of the loxP (P) sequence.

Recombinant ASFV viruses were constructed by homologous recombination between the MGF360 8*L* and MGF505 4*R* flanking regions of the transfected transfer vector DNA and the genome of the ASFV virus isolate BA71V within infected Vero cells (Fig. 1). Vero cells were infected with ASFV tissue culture adapted strain BA71V followed by transfection with the transfer vector pΔMGFloxPGUS. After 48 h the virus was harvested and used to infect fresh Vero cells and an overlay of 1% agarose applied. Seven days following infection an additional overlay containing 100 μg/ml X-Gluc was applied. Blue plaques containing ASFV recombinant viruses expressing GUS were picked into DMEM media. The frequency with which recombinant ASF viruses were generated by homologous recombination was found to be approximately one recombinant for every 5000 wild type virus progeny (data not shown). Plaque purification was repeated seven times until no further ‘white’ wild type plaques could be observed on plates. The isolated recombinant virus VΔMGF-GUS was grown into high titre stocks, and viral genomic DNA was isolated and analysed by PCR and DNA sequencing to confirm the insertion of the GUS marker gene and loxP sites and deletion of the truncated MGF360 9*L*, MGF505 2*R* and 3*R* genes (Fig. 2).

PCR analysis, using primers from within the virus genes MGF 360 8*L* and MGF505 4*R* flanking the insertion site, identified the expected the 3.2 kb fragment containing the truncated MGF360 9*L*, MGF505 2*R* and 3*R* genes in wild type virus BA71V (Fig. 2A lane 1). From genomic DNA of recombinant virus VΔMGF-GUS a 2 kb fragment was amplified, as expected, for the virus with MGF 505 2*R* and 3*R* genes replaced with the GUS marker gene cassette (Fig. 2A lane 2). To confirm that the recombinant virus VΔMGF-GUS contains the GUS gene, a PCR reaction was carried out with an internal GUS gene primer RGUS and primer 8LSEQ from within the MGF360 8*L* gene. The fragment amplified was the predicted 1.6 kb size (Fig. 2A lane 5). No PCR fragment was obtained with BA71V as template (Fig. 2A lane 4) as BA71V does not contain a GUS gene. To confirm that the GUS marker gene had been inserted at the correct site within the genome of recombinant VΔMGF-GUS, a PCR reaction was carried out with a primer, EXT8L, located in the MGF 360 8*L* gene but 5′ external to the left flank sequence of transfer vector VΔMGFloxPGUS. The results show that the recombinant virus VΔMGF-GUS (Fig. 2A lane 8) contains the predicted fragment of 2.2 kb inserted in the expected location in the genome. Analysis of samples from the BA71V parental genome (Fig. 2A lane 7) showed no products were amplified with primers EXTMGF and RGUS as was expected since BA71V does not contain the GUS gene.

### Removal of the GUS gene from recombinant VΔMGF-GUS using the Cre recombinase enzyme

To remove the GUS gene flanked by loxP sites from the recombinant VΔMGF-GUS (Fig. 1), a plasmid, pVP72iCre, was constructed. This contains the cre recombinase gene under the control of the ASFV B646L (vp72) gene promoter (see the Materials and methods section). After infection of Vero cells with recombinant VΔMGF-GUS, the cells were transfected with plasmid pVP72iCre. Three days after infection, the virus was harvested and screened for ‘white’ recombinant viruses using the indicator X-Gluc as before. The frequency with which the GUS gene was deleted by site-specific recombination by the Cre recombinase was determined to be approximately one recombinant progeny viruses for every ten ‘wild type’ virus (data not shown). This is a significantly higher recombination rate compared to the homologous recombination rate observed to delete the MGF505 2*R* and 3*R* genes. Following three rounds of plaque purification the recombinant virus VΔMGFΔGUS was grown up to high titre and genomic DNA isolated.

To confirm deletion of the GUS gene from recombinant VΔMGFΔGUS, PCR was used with virus genomic DNA as template. The results in Fig. 2A show that using primers from the flanking genes MGF360 8*L* and MGF 505 4*R* a fragment of about 0.23 kb was amplified (Fig. 2A lane 3). This was about 1.8 kb smaller than the 2.0 kb fragment amplified from the genome of VΔMGF-GUS virus (Fig. 2A lane 2) and confirmed that the GUS reporter cassette had been deleted as expected from VΔMGFΔGUS (Fig. 2A lane 3) compared to the parental virus VΔMGF-GUS (Fig. 2A lane 2). Additional confirmation that the GUS gene had been deleted from VΔMGFΔGUS is shown in Fig. 2A lanes 6 and 9, which show that no PCR fragment was amplified from the recombinant VΔMGFΔGUS using an internal GUS gene primer RGUS and the primers 8LSEQ, EXT8L, respectively.

**Fig. 3:**
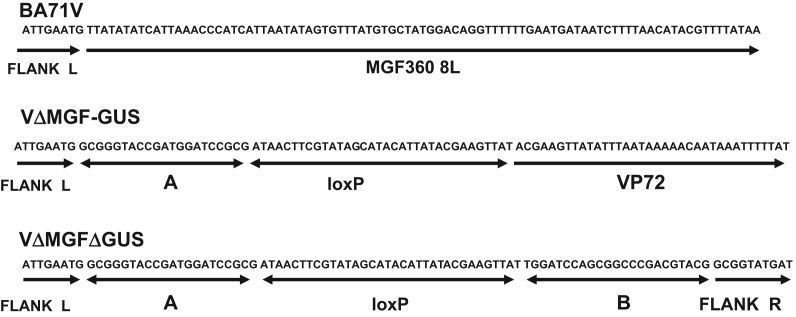
DNA sequence analysis of viruses BA71V, VΔMGF-GUS and VΔMGFΔGUS at insertion site. The primers 8LSEQ and 4RSEQ were used to amplify by PCR DNA fragments from genomic DNAs spanning the insertion and deletion junctions between the MGF360 8*L* and 9*L* and MGF 505 3*R* and 4*R* genes were cloned into the Hind*III* and Xho*I* sites of pCDNA3. The fragments were sequenced with the 8LSEQ primer. Non viral sequences A and B were derived from the transfer vector pΔMGFloxPGUS. The sequences underlined indicate the MGF360 8*L* sequence, loxP sequence and non-viral sequences A and B. Sequences of the flanking genes to the left and right (FLANK L and FLANK R) are also indicated.

To confirm the sequence at the sites of recombination within the recombinant viruses VΔMGF-GUS and VΔMGFΔGUS, DNA fragments were amplified by PCR from virus genomic DNA using the primers 8LSEQ and 4RSEQ from genes MGF360 8*L* and MGF 505 4*R* which flank the insertion sites. These fragments were cloned into the vector pCDNA3. Sequencing of the junctions (Fig. 3) revealed that in recombinant virus VΔMGF-GUS the GUS gene had been inserted preceded by the non-viral A sequence (21 bp), a loxP sequence (34 bp) and B646L (vp72) promoter (39 bp). In the recombinant derived from this virus VΔMGFΔGUS, analysis of the sequence confirmed that the GUS gene had been deleted along with one copy of loxP, leaving the sequence A (21 bp), loxP (34 bp) and sequence B (23 bp). Thus the sequencing confirmed that the Cre recombinase had removed the vp72GUS gene and one loxP site and that a single loxP site remained. Having established that the recombinant virus VΔMGF-GUS contained a loxP site flanking the 5′ end of the vp72GUS gene (Fig. 3) a PCR was carried out to confirm that a second loxP site flanked the 3′ end of the vp72 GUS gene. Primers from the GUS gene and MGF505 4*R* genes generated a predicted fragment of 141 bp in a PCR reaction confirming the presence of a 34 bp loxP site (data not shown).

### Deletion of the EP402R and EP153R genes from recombinant virus VΔMGFΔGUS

To confirm that a gene deletion could be made at a second locus on the BA71V genome using the GUS reporter gene, the same homologous recombination process was used to delete the EP402R and EP153R genes from virus VΔMGFΔGUS. This also allowed the effect of this combination of gene deletions on virus replication to be analysed. Since the EP402R and EP153R genes are adjacent on the ASFV genome, they were deleted together. Initially a transfer vector, pΔCD2v-LectinloxPGUS (the Materials and methods section), was constructed which contained the left and right flanking regions of the EP402R and EP153R genes from genes EP152R and EP364R, respectively. The GUS gene under control of the B646L ASFV gene late promoter and flanked by loxP sites and unique sites A and B was inserted between the flanking sequences as described in the previous section.

Vero cells were infected with the recombinant VΔMGFΔGUS and transfected with the transfer vector pΔCD2v-LectinloxPGUS. The progeny viruses were harvested and recombinant viruses expressing GUS were identified as forming blue plaques when X-Gluc was added in the agarose overlay. Recombinant virus VΔMGFΔCD2vLectin-GUS was plaque purified until no ‘white’ plaques could be observed. High titre stocks of virus were grown and viral genomic DNA was isolated.

PCR was carried out using primers from the flanking regions 152RSEQ and 364RSEQ. This amplified a 1.8 kb fragment from virus VΔMGFΔGUS which is the size predicted for the fragment containing the EP402R and EP153R genes (Fig. 2A lane 10). The fragment amplified from recombinant VΔMGFΔCD2vLectin-GUS was 2.0 kb, the size predicted for the deletion of the EP153R and EP402R genes and replacement with the GUS gene cassette (Fig. 2A lane 11). To confirm that the recombinant virus VΔMGFΔCD2vLectin-GUS contained the GUS gene a PCR reaction was carried out with an internal GUS gene primer, RGUS, and primer 152RSEQ. The results in Fig. 2 lane 14 show that the predicted fragment of 1.6 kb was amplified from the genome of recombinant virus VΔMGFΔCD2vLectin-GUS. To confirm that the GUS marker gene had been inserted at the correct site within the genome of recombinant VΔMGFDCD2vLectin-GUS a PCR reaction was carried out with primer EXT152R. This primer is located in the EP152R gene but is 5′ external to the left flank sequence of transfer vector pΔCD2v-LectinloxPGUS. The results show that the recombinant virus VΔMGFΔCD2vLectin-GUS (Fig. 2 lane 17) contains the predicted fragment of 2.8 kb. These primers did not amplify a fragment from the BA71V genome (Fig. 2A lane 16) as it does not contain a GUS gene.

### Removal of the GUS gene from recombinant VΔMGFΔCD2vLectin-GUS using the Cre recombinase protein

Vero cells were infected with recombinant VΔMGFΔCD2vLectin-GUS, and then transfected with plasmid pVP72iCre. Several days after infection, the virus was harvested from cell supernatants and screened for recombinant viruses from which the GUS gene had been deleted using the indicator X-Gluc as before. Plaques which were not blue were picked. Following three rounds of plaque purification the recombinant virus VΔMGFΔCD2vLectinΔGUS was grown up to high titre and genomic DNA was isolated.

To confirm deletion of the GUS gene from recombinant virus VΔMGFΔCD2vLectinΔGUS, PCR with primers 152RSEQ and 364RSEQ was carried out with genomic DNA as template. The results in Fig. 2A show that a fragment of 2 kb was amplified from the genome of the virus VΔMGFΔCD2vLectin-GUS (Fig. 2A lane 11) compared to a fragment of about 230 bp from the virus VΔMGFΔCD2vLectinΔGUS (Fig. 2A lane 12). Additional confirmation that the GUS gene has been deleted from VΔMGFΔCD2vLectinΔGUS is shown in Fig. 2A lanes 15 and 18, which show that no PCR fragment is amplified from the genome with the internal GUS gene primer RGUS and the two primers 152RSEQ and EXT152R, respectively.

Deletion of the EP153R and EP402R genes in virus recombinant VΔMGFΔCD2vLectin-GUS did result in changing the phenotype of the virus to haemadsorption (HAD) negative, rendering the virus unable to cause haemadsorption of pig red blood cells to Vero infected cells (data not shown). This was expected since the EP402R gene is essential for haemadsorption (Rodriguez et al., 1993; Borca et al., 1994).

**Fig. 4:**
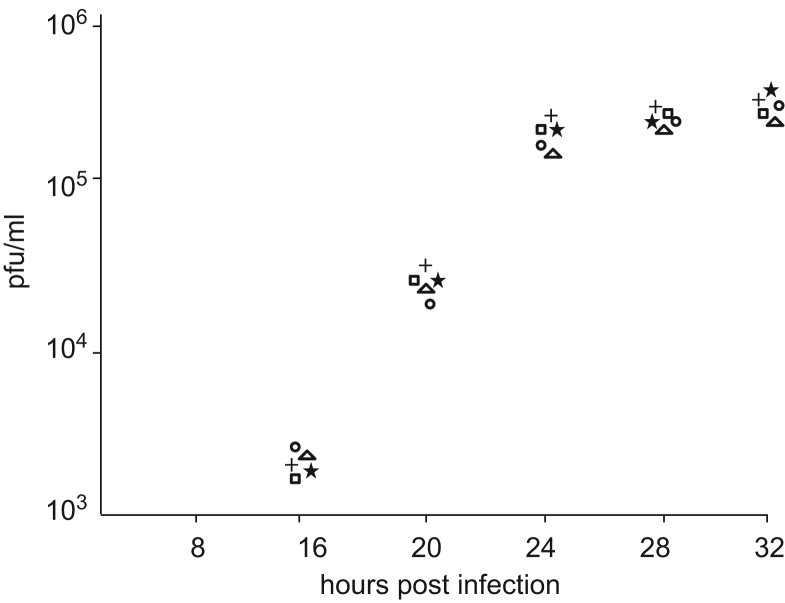
Replication kinetics of wild type BA71V and recombinant viruses. Vero cells were infected at a multiplicity of infection of 10 with parental BA71V strain or recombinant viruses VΔMGF-GUS, VΔMGFΔGUS, VΔMGFΔCD2vLectin-GUS, VΔMGFΔCD2vLectinΔGUS. At various hours post-infection, as indicated on the *x* axis, total virus was harvested and infectious virus titrated by plaque formation on Vero cells. The virus titre shown is the mean of triplicate samples. The titre obtained is indicated in plaque forming units per ml (pfu/ml) on the *y* axis. Titres obtained following infection with viruses BA71V □, VΔMGF-GUS ○, VΔMGFΔGUS +, VΔMGFΔCD2vLectin-GUS Δ and VΔMGFΔCD2vLectinΔGUS ⋆ are indicated.

The ability of the different recombinant ASF viruses, VΔMGF-GUS, VΔMGFΔGUS, VΔMGFΔCD2vLectin-GUS, VΔMGFΔCD2vLectinΔGUS to replicate in Vero cells was, compared with the parental BA71V strain by conducting a one step growth curve. Vero cells were infected at high m.o.i. (10 pfu/cell) and titres of total infectious progeny virus were determined at different time points post-infection. The results in Fig. 4 showed that the four recombinant viruses all replicated with similar kinetics and reached approximately the same titre as wild type BA71V. This showed that deletion of the truncated MGF360 9L, MGF505 2R and 3R genes and EP153R and EP402R genes or the insertion of the GUS marker gene flanked by loxP sequences do not significantly alter the replication fitness of these recombinant viruses.

ASFV encodes a large number of genes which are not essential for virus replication and these include genes encoding proteins that modulate host defence pathways (Tulman et al., 2009). In common with other large DNA viruses a number of proteins have been described which target the same pathways, for example several apoptosis inhibitors and more than one inhibitor of phosphorylation of translation initiation factor eIF-2α have been identified (Zhang et al., 2010) and several inhibitors of apoptosis (Tulman et al., 2009). In order to understand the importance of these pathways for virus replication a method which enables sequential deletions of genes from the virus genome is required. This is also an important tool for the construction of rationally attenuated ASFV vaccines.

Here we describe a method to sequentially delete genes from the ASFV genome making use of the cre/loxP recombination system to remove the GUS marker gene from first generation ASFV recombinants so that the same marker can be used for additional deletions. Using this, a recombinant ASFV virus VΔMGF-GUS was constructed in which the MGF505 genes 2*R* and 3*R* and a truncated version of MGF360 9*L* genes were first deleted with the introduction of the GUS marker gene flanked by bacteriophage loxP sites. The GUS reporter gene was then deleted by site–specific recombination at the two loxP sites. This recombination was achieved at high efficiency (approximately 10% of virus progeny). Subsequently the same marker cassette was used to isolate recombinant virus VΔMGFΔCD2vLectin—GUS with the EP153R and EP402R genes deleted in addition to the previous deletion.

The cre/loxP site-specific recombination system from bacteriophage P1 (Sternberg and Hamilton, 1981) results in deletion of DNA sequence located between two loxP sites oriented in the same direction by the bacteriophage Cre recombinase. The 34 bp loxP sites consist of an asymmetric 8 bp sequence flanked by two identical palindromic 13 bp sequences. The 343 amino acid Cre recombinase protein belongs to the integrase family of enzymes isolated from λ bacteriophage P1. This site-specific recombination system was first used in mammalian cells by Sauer and Henderson (1988). The cre/loxP bacteriophage recombination system has been used previously to create HSV recombinant viruses (Gage et al., 1992). However, these recombinant viruses were initially generated in a cell free environment by Cre recombinase induced integration of loxP containing plasmids into the purified DNA genome of HSV, followed by the transfection of genomic DNA into Vero cells before the isolation of HSV recombinant viruses. This method for generating recombinant viruses in ASFV could not be used as ASFV genomic DNA is not infectious.

In this study a truncated gene MGF360 9*L* and two complete MGF505 genes, 2*R* and 3*R* were deleted from the genome of the tissue-culture adapted ASFV isolate BA71V. BA71V already has a large deletion of 8 kb near the left end of the genome compared to virulent ASFV isolates and lacks 5 complete members of MGF360 and 1 member of MGF505 encoded in this fragment by virulent isolates (Chapman et al., 2008).

Previous studies have indicated that members of MGF360 and MGF530 gene families have roles in macrophage cell tropism, suppression of the type I interferon response and replication of the virus in *Ornithodoros spp*. ticks (Zsak et al., 2001; Neilan et al., 2002; Afonso et al., 2004; Burrage et al., 2004). The protein encoded by the EP402R gene, the CD2v protein, is required for haemadsorption of red blood cells to infected macrophages and extracellular virions (Rodriguez et al., 1993), and has roles in inhibiting proliferation of lymphocytes in response to mitogens and virus replication in ticks (Borca et al., 1998; Rowlands et al., 2009). The EP153R protein is a C-type lectin domain containing protein which resembles NK cell receptors and has been reported to inhibit up-regulation of MHC-Class I expression on infected cells and inhibit apoptosis (Hurtado et al., 2011). Deletion of these MGF505 2*R* and 3*R* genes in recombinant virus VΔMGF-GUS and the further deletion of the EP153R and EP402R genes in recombinant virus VΔMGFΔCD2vLectin-GUS did not significantly alter the growth characteristics of these viruses compared to wild type BA71V.

In this paper we have described the efficient sequential deletion of specific genes from the genome of tissue culture adapted strain BA71V using the bacteriophage cre/loxP recombination system. In the future, this technology could be applied to manipulate the genomes of field strain ASF viruses to create attenuated vaccine strains of ASFV. The generation of recombinant ASF field viruses with multiple gene deletions will be achieved by the infection and transfection of isolated pig bone marrow macrophages in vitro. In addition, the cre/loxP technology could be used for the efficient introduction of mutated ASFV genes into the ASFV genome in order to study individual gene function.

## Materials and methods

### Viruses and cell culture

Vero cells were grown in Dulbecco's modified Eagle's media (DMEM) containing 10% FCS, glutamine 2 mM, penicillin and streptomycin (10,000 u mg^−1^ml^−1^). Tissue culture adapted ASFV strain BA71V and the recombinant ASF viruses VΔMGF-GUS, VΔMGFΔGUS, VΔMGFΔCD2vLectin-GUS and VΔMGFΔCD2vLectinΔGUS were propagated and titrated on Vero cells (Enjuanes et al., 1976).

### Construction of plasmid transfer vectors

The plasmid transfer vector pΔMGFloxPGUS was constructed to facilitate the deletion of genes MGF505-2*R* and MGF505-3*R* from the genome of virus BA71V as shown in Fig. 1. Using BA71V genomic DNA as template, *a* 727 bp fragment (Flank L) located in the MGF360 8*L* gene at position 16,351–17,056 upstream of the truncated MGF 360 9*L* gene and MGF505-2*R* gene was amplified using the PCR primers LFMGF (GCGCAAGCTTGCTATGGCATACCAGTATTTAACG) and LRMGF (CGCGGATCCATCGGTACCCGCCATTAATATATGGATTGACATG). This left hand flank fragment also contained a non viral sequence A at the 3′ end to facilitate further overlapping PCR. A 681 bp fragment (Flank R) located in the MGF505 4*R* gene at position 20,236–20,897 downstream of the MGF505 3*R* gene was amplified using the PCR primers RFMGF (TGGATCCAGCGGCCCGACGTACGGCGGTATGATGTACCCAATTGTAATG) and RRMGF (GCGCCTCGAGGCCATCTCAAACAATTCCTGATTCTCTCCC). This right hand flank fragment also included a non-viral sequence B at the 5′ end. Two GUS gene fragments AloxPvp72GUS (1390 bp) and GUSloxPB (680 bp) containing the N- and C-terminal gene fragments, respectively, were amplified from GUS gene template using the primer pairs ALOXPGUS (GCGGGTACCGATGGATCCGCGATAACTTCGTATAGCATACATTATACGAAGTTATATTTAATAAAAACAATAAATTTTTATAACATTATAT) and RGUS (CCTTCTCTGCCGTTTCCAAATCGCCGC); FGUS (CGCGCCACTGGCGGAAGCAACGCG) and BLOXGUS (ACTTACGTCGGGCCGCTGGATCCATAACTTCGTATAATGTATGCTATACGAAGTTATCATTGTTTGCCTCCCTGCTGCGGTTTTTCAC). The AloxPvp72GUS fragment contained the ASFV B646L gene promoter sequence fused upstream of the GUS gene start codon and upstream of this a single loxP sequence and the unrelated sequence A to facilitate overlapping PCR amplification. The GUSloxPB fragment contained a single loxP site, in the same orientation as that in the AloxPvp72 GUS fragment, and an unrelated sequence B, downstream from the GUS gene stop codon. In the second stage, overlapping PCR was used to generate two separate DNA fragments. The first FlankLAloxPvp72GUS (2219 bp) was amplified by PCR from templates Flank L and AloxPvp72GUS using primer pair LFMGF and RGUS. The second fragment GUSloxPBFlankR was amplified from the PCR templates GUSloxPB and FlankR using primer pair FGUS and RRMGF. A final overlapping PCR reaction was carried out to amplify the DNA fragment FlankLAloxPvp72GUSloxPBFlankR using the templates FlankLAloxPvp72GUS and GUSloxPBFlankR and the primer pair LFMGF and RRMGF. The 3.4 kb amplified fragment was digested with *Hind* III and *Xba* I enzymes and cloned into pCDNA3 plasmid vector digested with the same enzymes to create transfer vector pΔMGFloxPGUS. The structure of the plasmid pΔMGFloxPGUS was confirmed by restriction enzyme digestion (data not shown).

The plasmid transfer vector pΔCD2v-lectinloxPGUS was constructed to delete the EP402R and EP153R genes, encoding the CD2v and C-type lectin proteins. The same method as above was used to construct the transfer plasmid pΔCD2v-LectinloxPGUS except different primers sets (LFCD2v GCGCAAGCTTCCTTGTTTTATTTATTATTATATACACCGC and LRCD2v CGCGGATCCATCGGTACCCGCGATTAATTTTGTGTTATATATTTTTCAACCG) (RFCD2v TGGATCCAGCGGCCCGACGTACGTCATGTATTTATTAAATACCACG and RRCD2v GCGCCTCGAGCGCACACCATTTCCTTAGAAAAGCC) were used to generate the upstream left flank and downstream right flank fragments, respectively.

### Construction of plasmid expressing Cre recombinase pVP72iCre

The 1070 bp DNA fragment containing the B646L gene promoter sequence, ATTTAATAAAAACAATAAATTTTTATAACATTATAT, upstream of the Cre recombinase gene was synthesised (Geneart, Germany). The sequence of the cre recombinase gene was based on the synthetic construct Pubmed Accession Number AY056050 but the nuclear localisation site was removed and the codon usage optimised for mammalian gene expression. The 1070 bp DNA fragment was digested with enzymes *KpnI* and *SacI* and following filling of the ends to make them blunt-ended, was cloned into the pCDNA3 vector digested with *EcoRV* to create pVP72iCre.

### Construction and isolation of recombinant ASF viruses

Vero cells (35 mm dish) at 75% confluency were infected with BA71V at a multiplicity of infection (m.o.i). of 10 and incubated at 37 °C for 5 h, and then washed with Dulbecco's minimal essential medium (DMEM). A transfection mixture containing 250 μl Optimem (Gibco), 5 μg DNA transfer vector pΔMGFloxPGUS or pΔCD2v-LectinloxPGUS and 7.5 μl TRANS-IT (Mirus) transfection reagent was incubated at 20 °C for 20 min before adding it to the infected cells. Incubation was continued at 37 °C for 4 h before the addition of 1 ml DMEM and continued incubation at 37 °C. Virus was harvested from the infected and transfected cells 48 h post-infection. The harvested virus was used to infect 50% confluent Vero cells (100 mm dish) at a m.o.i. of 0.005 at 37 °C. Four hours after infection the virus inoculum was removed and cells were washed once in DMEM then a 1% Agarose/DMEM overlay containing 2% FCS was applied. Five days after infection a second overlay containing 1% agarose and 100 μg/ml X-Gluc (5-bromo-4-chloro-3-indolyl-*b*-d-glucuronic acid) was applied to the infected cells and recombinant ASFV viruses were identified by the ‘blue’ appearance of the plaques. Individual blue plaques were picked into 1 ml DMEM and recombinant viruses were subjected to a further seven additional plaque purifications until no ‘white’ plaques containing wild type viruses could be observed. For removal of the GUS reporter gene construct using cre recombinase, Vero cells were infected with recombinant viruses expressing the GUS gene and transfected with plasmid pVP72iCre expressing the Cre recombinase. Progeny virus was harvested from culture supernatants after 3 days and used to infect Vero cells. Recombinant viruses from which the GUS gene had been excised were identified as “white” plaques in the presence of overlay containing X-Gluc. and were purified by plaquing until no “blue” plaques were observed.

### Purification and analysis of viral genomic DNA

Viral genomic DNA was purified from 300 μl virus containing supernatants from infected cells using a GE Healthcare Illustra genomic Prep Mini Spin kit. Analysis of viral genomic DNA was carried out by PCR using the specific DNA primers 8LSEQ (CATGGTATAGAGAATATCATG), 4RSEQ (GATGTGGTCAAATAGTTAAG), EXT8L (GATAGAAAAAGAAGATAGCTC), RGUS (CCTTCTCTGCCGTTTCCAAATCGCCGC), 152RSEQ (CGCCACCGCACTAGGAAAAACGGTTG), 364RSEQ (GAAGACCAGCTTGAAACG) and EXT152R (GGTTGTGATGGTGCATGTAAC).

To confirm the DNA sequence at the junction of recombination sites, fragments of genomic DNA from wild type and recombinant viruses were amplified using primers 8LSEQ and 4RSEQ and cloned into plasmid pCDNA3 at the *HindIII* and *XhoI* sites. The recombinant DNA junctions were sequenced using primer 9LSEQ on an ABI Prism 373 DNA Sequencer and analysed using Chromas software.
